# The healing power of transcutaneous electrical nerve stimulation: a systematic review on its effects after breast surgery

**DOI:** 10.1007/s00520-024-09129-3

**Published:** 2025-01-13

**Authors:** Seda Akutay, Hatice Yüceler Kaçmaz, Özlem Ceyhan

**Affiliations:** 1 Department of Surgical Nursing, Faculty of Health Sciences, Erciyes University, Kayseri, Turkey; 2 Department of Internal Nursing, Faculty of Health Sciences, Erciyes University, Kayseri, Turkey

**Keywords:** Breast cancer, Transcutaneous electrical nerve stimulation, Pain, Postoperative care

## Abstract

**Background:**

Transcutaneous electrical stimulation after breast cancer surgery has been utilized for various purposes, but the full efficacy of this treatment approach on postoperative symptoms remains unclear.

**Aim:**

This study aimed to answer the question: Does transcutaneous electrical nerve stimulation significantly impact postoperative patient outcomes in individuals undergoing breast cancer surgery?

**Methods:**

A systematic review of randomized controlled trials was conducted. Because of the limited number of studies included, it was not feasible to perform a meta-analysis. English-language publications from 2013 and 2024 that investigated the effects of transcutaneous electrical stimulation in breast cancer surgery patients were included. Electronic databases such as Web of Science, PubMed, Scopus, EBSCO, ScienceDirect, Cochrane Central Register of Controlled Trials, and Wiley Online Library were searched. Two independent investigators assessed the studies using the revised JBI risk of bias tool. Data from randomized trials were extracted by two researchers using the Cochrane data collection tool.

**Results:**

Our comprehensive literature review identified 251 studies. After rigorous assessment, 12 articles met our inclusion criteria. Title and abstract screening excluded seven studies that did not involve surgery, used treatments other than TENS, included acupuncture, or did not measure pain outcomes. Among these, five studies involving 776 patients examined the effects of transcutaneous electrical stimulation on pain management in breast cancer surgery. In all of the studies reviewed, transcutaneous electrical stimulation had a beneficial effect on postoperative pain.

**Conclusion:**

Transcutaneous electrical stimulation has significantly alleviated pain associated with breast cancer surgery. This therapeutic modality has improved patient satisfaction with analgesia by relieving pain; reducing analgesic use; reducing postoperative nausea and vomiting; increasing blood levels of IL-2, IFN-γ, and IL-2/IL-4 ratio; and reducing skin sensitivity. Transcutaneous electrical stimulation devices may improve postoperative patient outcomes and enhance the recovery process in people undergoing breast cancer surgery. The results of this study are limited by heterogeneity and the small number of included studies. Future research should prioritize standardization of intervention procedures and investigation of the long-term effects of TENS in postoperative care.

**Registration:**

This study was registered in the PROSPERO registration system under the number CRD42024523558.

**Supplementary Information:**

The online version contains supplementary material available at 10.1007/s00520-024-09129-3.

## Introduction

Breast cancer is the most commonly diagnosed cancer worldwide, accounting for approximately 12% of all cancer cases [1]. Surgical treatment is a crucial aspect of breast cancer treatment, involving either partial or complete removal of breast tissue depending on the cancer spread and tumor size [2, 3]. Patients undergoing breast surgery often experience symptoms and psychosocial issues such as postoperative pain, decreased quality of life, lymphedema, arm numbness, stiffness, and limited shoulder range of motion [4]. A multidisciplinary approach that considers physical, psychological, social, and vocational rehabilitation is necessary to maintain and enhance patients’ quality of life. Promoting early functional recovery and prevention complications are crucial to accelerating the recovery process and help individuals reintegration into daily life without limitations. A combination of different methods is recommended for postoperative care following breast cancer surgery to achieve these goals [5].

Transcutaneous electrical nerve stimulation (TENS) is a non-pharmacological treatment method known for its enhanced analgesic effect after mastectomy [6–8]. TENS has peripheral and central mechanisms that contribute to the reduction of hyperalgesia. The peripheral mechanism of TENS produces an analgesic effect by reducing the excitability of nociceptors in the application area. High-frequency TENS reduces substance P levels, while low-frequency TENS acts through peripheral opioid and adrenergic receptors and can increase blood flow. The central mechanism of TENS involves the formation of a complex network of neurons to reduce pain. TENS activates large-diameter afferent fibers, such as A-beta fibers, which inhibit nociceptive signals at the spinal cord level through the gate control mechanism. This input is then sent to the central nervous system and triggers inhibitory systems that reduce hyperalgesia [9, 10].

The Transcutaneous Electrical Acupuncture Stimulation (TEAS) method is a contemporary therapeutic modality that combines the stimulation of acupuncture points with transcutaneous electrical nerve stimulation (TENS). The fundamental principle of TEAS is the stimulation of sensory nerve endings along acupuncture meridians through the application of low-voltage electrical current. This modality exerts its biological effects through various molecular pathways, including the release of endogenous opioids [11, 12], which may inhibit pain transmission [13], improve local circulation [14], and enhance immunity [6]. Preoperative application of TEAS has been associated with better quality of life, reduced pain severity, and increased patient satisfaction [15]. However, more research is needed to determine the efficacy and safety of TEAS for postoperative pain management [16]. However, strong clinical evidence considering the effects of TENS or TEAS in the assessment of patient outcomes after breast cancer surgery is still lacking.

This study aimed to systematically review recent literature on transcutaneous electrical stimulation methods (TENS and TEAS) in patients undergoing breast cancer surgery, with a focus on postoperative outcomes.

## Methods

### Study design

This study is a systematic review of randomized controlled trials conducted in accordance with the Preferred Reporting Items for Systematic Reviews and Meta-Analyses guidelines (PRISMA) [17].

### Literature search

In this study, we searched electronic databases, including Web of Science, PubMed, Scopus, EBSCO, ScienceDirect, Cochrane Central Register of Controlled Trials, and the Wiley Online Library between March and April 2024, using the keywords provided in Table 1. We combined the keywords with boolean operators “AND” and “OR.” Two researchers independently conducted the literature search, with the third researcher consulted in cases of disagreement. This study was registered in the PROSPERO (protocol number: CRD42024523558).

**Table 1:** Search strategy

Search	Keywords
#1	“mastectomy” OR “breast neoplasms” OR “breast cancer” OR “breast cancer surgery” OR “breast conserving therapy”
#2	“TENS” OR “Transcutaneous Electrical Nerve Stimulation” OR “electric stimulation” OR “Electrotherapy” OR “transcutaneous electrical acupoint stimulation” OR “TEAS”
#3	“Randomized control trial” OR “Randomized controlled trial” OR “Randomized” OR “controlled clinical trial” OR “RCT”
#4	#1 AND #2 AND #3

### Inclusion criteria

The PICOS (P, population; I, interventions; C, comparators; O, outcomes; S, study designs) approach was used to formulate the research question and identify keywords. Study participants (P) were patients older than 18 years undergoing surgery for breast cancer. Interventions (I) included transcutaneous electrical nerve stimulation (TENS) and transcutaneous electrical acupoint stimulation (TEAS) therapies applied to cancer patients. TENS and TEAS were included as search terms to ensure a comprehensive review of both techniques. While similar in employing electrical stimulation, TENS and TEAS differ in application points and mechanisms. The control (C) group received standard care, placebo, and sham TENS methods. The outcome variables of the study included symptoms. The primary outcome is pain symptom. Secondary outcomes included patient satisfaction, reduced analgesic consumption, alleviated postoperative nausea and vomiting, preserved cellular immune function, and relieved skin sensitivity. The studies included in this systematic review were randomized controlled trial (RCT) designed (S) studies. When assessing the effects of interventions, non-randomized studies are likely to have more potential bias than randomized studies, so only randomized controlled trials were considered in this study [18]. According to the PICOS strategy, the research question was “Does transcutaneous electrical nerve stimulation significantly impact postoperative patient outcomes in individuals undergoing breast cancer surgery?” Studies that met at least 1 of the following criteria were excluded:

1. Written in languages other than English. This decision was necessary to maintain methodological consistency and ensure accurate interpretation of results
2. Abstracts/posters
3. Published in non-peer-reviewed journals
4. Dissertations, letters, committee reports, conference proceedings, short articles, and expert opinions
5. Systematic reviews
6. Using non-electrical stimulation strategies

In this study, only randomized controlled trial articles were considered. Only studies published in English between 2013 and 2024 were included specifically; studies focusing on applying electrical stimulation to patients after breast surgery were included.

### Quality assessment

The Joanna Briggs Institute (JBI) quality assessment tool developed for randomized controlled trials was utilized to assess the studies’ quality [19]. The JBI Quality Assessment tool for RCTs comprises 13 items, each rated as “yes,” “no,” “uncertain,” or “not applicable.” This checklist focuses on risks of selection, performance, detection, and attrition bias regarding internal and external validity. The bias risk assessment was conducted independently by two researchers and reviewed by a third researcher in case of any disagreements during the assessment.

### Data extraction and synthesis

After completing the search in the databases, the studies to be included were identified by removing duplicate publications using a program (https://www.rayyan.ai/). Two independent researchers conducted data extraction. In addition to the study design, sample size, participant characteristics, details of the electrical stimulation, application protocols, primary and secondary outcome measures, effect size with 95%CI, and side effects were also extracted.

### Data analysis

After completing the data extraction process from the studies that met the inclusion criteria, the effect size was calculated using Hedge’s g in the Comprehensive Meta-Analysis (CMA) (version 4) software to evaluate the data’s suitability for meta-analysis. The random effects model was initially employed for analysis. The small number of included studies and their significant methodological heterogeneity limited the feasibility of a meta-analysis. Instead, effect size (ES) is reported for primary and secondary outcomes in individual studies. The effect sizes were categorized according to Cohen’s *d* as small (0.2–0.5), medium (0.5–0.8), or large (> 0.8) and were provided with 95% confidence intervals (95% CI). A significance level of *p* < 0.05 was considered for all analyses.

## Results

### Study selection

**Fig. 1:**
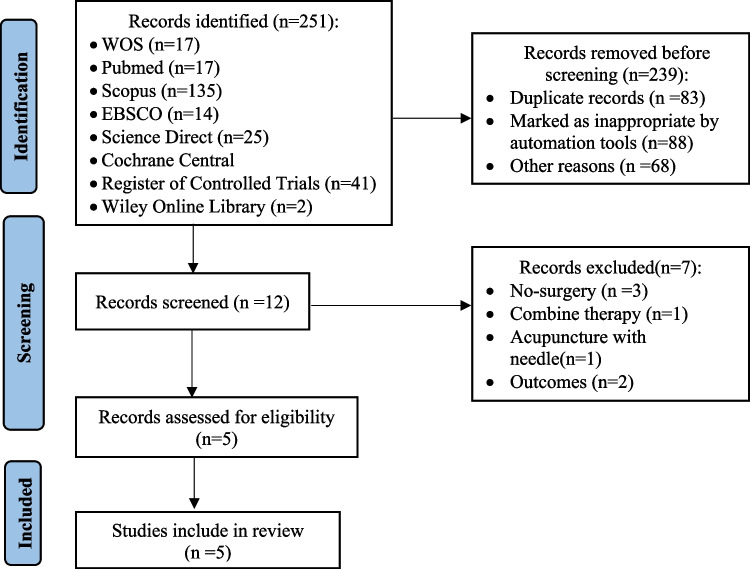
PRISMA 2020 flow chart

Two hundred fifty-one articles were found due to the search strategy. After removing duplicate publications (*n* = 83), 12 studies that met the inclusion criteria [5–7, 20–28] were evaluated in terms of eligibility. When the titles and abstracts of these studies were scanned, studies that did not include surgical intervention, applied other treatments in addition to TENS therapy in the intervention group, applied needle acupuncture, and did not include pain outcome parameters were excluded. It was decided to include five articles in the review (Fig. 1).

### Study characteristics

**Fig. 2:**
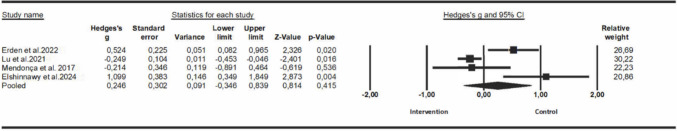
Comparison of pain intensity between intervention and control groups according to the VAS scale

The included studies were published between 2017 and 2024. The studies addressed the effects of TENS and TEAS in the management of pain after breast surgery in a total of 776 patients. Included were randomized controlled trials from Brazil [5], China [6, 23], Turkey [7], and Egypt [21]. The sample size of the studies varied between 30 and 568 (mean = 155). In all of the studies, pain intensity was the primary outcome parameter, and besides analgesic consumption, endotracheal intubation time, patient satisfaction, sleep quality, postoperative nausea-vomiting, perioperative immunity, skin sensitivity, and nerve conduction were evaluated. Except for one study [16], pain intensity was assessed according to VAS in all studies and the effect sizes were statistically significant at 0.52 (95% CI 0.08 to 0.97) medium effect [7], − 0.25 (95% CI − 0.45 to − 0.05) small effect [23], and 1.10 (95% CI 0.35 to 1.85) large effect [21], respectively. In Mendonça et al. study, the ES was − 0.21 (95% CI − 0.89 to 0.46) small effect [5], and not statistically significant. The study by Ao et al. calculated the ES based on the frequency of analgesic consumption and found it to be 1.46 (95% CI 0.91 to 2.01) large effect [16] (Table 2). The common ES of the studies [5, 7, 21, 23] according to pain intensity was determined to be a small effect of 0.25 (95% CI − 0.35 to 0.84) (*p* > 0.05) and was not statistically significant (Fig. 2).

**Table 2:** Characteristics of included studies

Author, year (country)	Study design	Sample size	Participant characteristics	Inclusion criteria	Randomization and blinding	Analgesia application	Outcomes	Effect size with 95% CI (*p* value)	Conclusion
Mendonça, 2017 [5] (Brazil)	Double-blinded RCT	33 participants (TENS group: 16, Placebo group:17)	Breast cancer patients undergoing axillectomy	• More than three months of reported increased cutaneous sensitization in the intercostobrachial nerve (ICBN) dermatome • No other underlying systemic disease • Never received a TENS application before • No TENS contraindications • Not using antidepressants, psychoactive drugs and glucocorticosteroids	Patients were randomly divided into two groups. This procedure ensured the study was double-blind (neither the patient nor the researchers knew which treatment was being administered)	NS	• Pain intensity • Quality of life • Score of PONV	− 0.21 (95% CI − 0.89 to 0.46) (*p* > 0.05)	Although similar to placebo, according to the methods used in the study, TENS was able to reduce the intensity of dysesthesia in the ICBN dermatome after breast cancer surgery but did not improve quality of life. Using a method with a short active stimulation may have potentiated placebo effects. These results may help clinicians and researchers approach oncology patients with altered skin sensitization. However, since the physical and sensory functionality of the limb is vital for women to perform their daily activities, new protocols or different therapeutic electrical currents could be used in the search for positive repercussions on quality of life
Ao, 2021 [6] (China)	RCT	65 participants (TEAS group: 32, Sham group: 33)	Breast cancer patients undergoing radical mastectomy	• Elective radical mastectomy between 20 and 65 years of age • Women with ASA I-II score • BMI < 30 kg/m^2^ • Severe cardiac and respiratory problems, renal, hepatic, or immunodeficiency, history of immunosuppressive therapy (chemotherapy-radiotherapy) • Never received acupuncture/TENS therapy before • No history of chronic pain • No steroid or opioid use • No alcohol and substance addiction Patients without elevated CRP and leukocyte levels in previous surgery and without systemic infection at the application site infection	Participants were randomly allocated to the TEAS and sham groups using sequentially numbered sealed envelopes and a random number generator. The envelopes were prepared and distributed by an assistant not involved in this study. The relevant interventions were performed by an experienced acupuncturist independent of this study. The same group of surgeons performed all surgeries. Similarly, a blinded anesthesiologist provided anesthesia administration and perioperative care. A second anesthesiologist, blinded to the treatment regimen and not included in the data analysis, performed anesthesia follow-up	Participants in each group were provided with a patient-controlled analgesia (PCA) pump for postoperative pain control, and this pump was used for 48 h postoperatively. The PCA consisted of 1.5 µg/kg sufentanil diluted to 150 ml with normal saline. The basal infusion rate was set at 2 ml/h, the bolus dose was at 0.5 ml, and the lockout interval was 15 min	• Perioperative immunity • Pain intensity • Analgesic consumption	1.46 (95% CI 0.91 to 2.01)	This study provided a new perspective for postoperative analgesia selection by demonstrating that TEAS preserves cellular immune function, alleviates postoperative pain, and reduces the occurrence of opioid-related side effects. The results of this study may have implications for postoperative pain management in patients with cancer in terms of immune function and postoperative recovery
Erden, 2022 [7] (Turkey)	Prospective RCT	80 participants (TENS group: 40, Control group: 40)	Patients with breast cancer undergoing modified radical mastectomy (TENS group: 67.5% stage 3, Control group: 72.5% stage 3)	• Over 18 years old • No history of pacemaker and arrhythmia • No history of chronic pain and alcohol/substance abuse • No history of preoperative TENS or opioid use • No postoperative epidural analgesia • No cognitive impairment • No thoracic incision other than mastectomy incision • At least one drainage available • No metastatic disease • Those who do not need mechanical ventilation • Non-contraindications for TENS at the discretion of the treating doctor • No postoperative complications • American Society of Anesthesiologists (ASA) Class I-III • Those who volunteered to participate in the study	Patients were randomly assigned to TENS or control group (1:1). A random number generator and sequentially numbered, opaque, sealed envelopes were used. The envelopes were prepared by an assistant not involved in the current study. The nurse administering the data collection forms was blinded to the groups	The routine analgesic protocol (diclofenac 75 mg intramuscularly on postoperative day 0, paracetamol 500 mg tablet twice on postoperative day 1, and paracetamol as needed until discharge) was used in both groups	• Pain intensity • Patient satisfaction • Score of PONV	0.52 (95% CI 0.08 to 0.97) (*p* < 0.05)	TENS has been shown to reduce pain after mastectomy. The positive effects of TENS on pain control and improved physical and psychological outcomes make TENS a useful analgesic modality in this patient population
Elshinnawy, 2024 [21] (Egypt)	RCT	30 participants (TENS group:15, Cold application: group:15)	Breast cancer patients undergoing mastectomy	• 45–60 years • Chemotherapy-associated diabetic peripheral neuropathy after mastectomy surgery two years after chemotherapy With Type 2 Diabetes patients with an HbA1C value between 6–6.5	Patients were randomly divided into two groups of equal size. There is no mention of blinding	NS	• Pain intensity • Nerve amplitude	1.10 (95% CI 0.35 to 1.85) (p < 0.01)	This study shows that TENS is safe and effective in the treatment of symptomatic patients with post-mastectomy diabetic peripheral neuropathy. It was also shown that cold application was more effective than TENS in reducing pain in patients with post-mastectomy diabetic peripheral neuropathy
Lu, 2021 [23] (China)	Multi-center RCT	568 participants (Single acupoint TEAS group: 196, Combine acupoint TEAS group: 188, Sham group: 184)	Breast cancer patients undergoing radical mastectomy	• Elective radical mastectomy under general anesthesia • 18–65 years • Without TENS contraindications (scarring or skin damage at the stimulation site, electronic devices such as pacemakers or other medical electronic devices implanted in the body) • No communication difficulties • No history of breast surgery • No drug abuse/dependence or alcohol abuse or dependence • Heart dysfunction or severe hypertension without confirmed liver/renal dysfunction	Study participants were randomized 1:1:1 to sham surgery, single acupoint (TEAS at PC6), and combined acupoint groups (TEAS at PC6 and CV17) using a secure web-based system stratified by treatment center according to permuted blocks (randomized block sizes; maximum 12). Outcomes were assessed by trained investigators blinded to treatment allocation. Patients and investigators participating in the intervention were not masked. Other clinical staff, including surgeons, anesthesiologists, and ward staff, were unaware of the allocation	Sufentanil was used for postoperative patient-controlled analgesia. Parecoxib sodium (40 mg) was administered as rescue analgesia to patients with NRS higher than 4	• Pain intensity • Analgesic consumption • Score of PONV • Time to extubation • Time to a verbal response • Patient satisfaction	− 0.25 (95% CI − 0.45 to − 0.05) (*p* < 0.05)	In patients undergoing radical mastectomy under general anesthesia, stimulation to combined acupuncture points before induction of anesthesia is associated with less chronic pain 6 months after surgery than sham intervention In addition, combined acupoint stimulation is associated with lower analgesic consumption and lower postoperative nausea and vomiting scores compared to sham intervention

*CI* confidence intervals, *RCT* randomized controlled trial, *TG* TENS group, *CG* control group, *CAG* cold application group, *PG* placebo group, *PONV* postoperative nausea and vomiting, *NS* not specified

### Methodological quality

The risk of bias assessment for the five included studies is described in Table 3. One study had low overall quality and was at high risk of bias related to intervention/exposure management and selection and allocation [21]. In this study, although it is stated that the participants were randomly assigned to the intervention and control groups, the randomization method is not mentioned. Therefore, the selection and allocation process remains unclear [21]. In two of the studies, the allocation of participants to intervention and control groups was not concealed [21, 23]. Only one of the included studies was participants blinded to treatment assignment [5]. It was observed that participants and those delivering treatment blinding was not performed in most studies [6, 7, 21, 23]. Only one of the reviewed studies was outcome assessors blinded to treatment assignment [21].

**Table 3:**
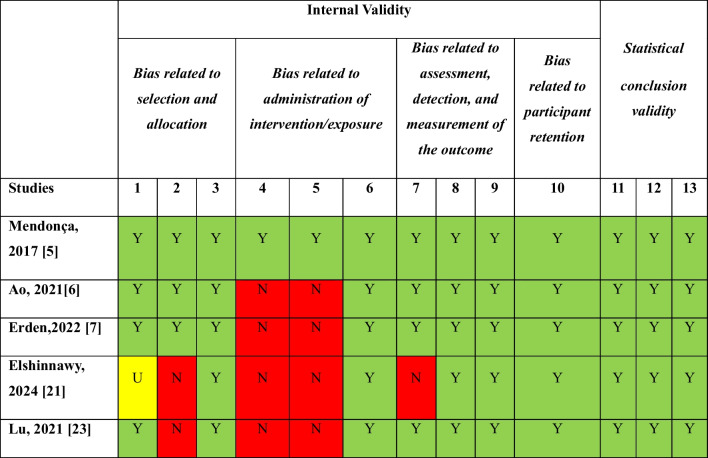
Critical appraisal checklist for randomized controlled studies

Green indicates yes, red signifies no, and yellow represents unclear

### Characteristics and application of TENS therapy

In one of the reviewed studies, electrodes were applied around the drain in the surgical field [7], in three studies to acupuncture points on the arms or legs [5, 6, 23], and in one study to acupuncture points on the medial knee and medial malleolus [21]. During TENS application, acupuncture points were stimulated—intensely in two of the studies [6, 23] and biphasically and asymmetrically in one study [5]. The waveform used in the two studies was not specified [7, 21]. In the study by Erden et al. [7], a high frequency of 85 Hz was used, while a low frequency of 2 Hz and 10 Hz for two cycles was used in the study by Lu et al. [23]. In the study by Ao et al., a burst-type frequency of 2–100 Hz was used [6]. In the study by Elshinnawy et al., a low frequency of 15 Hz was used [21], and a high frequency of 100 Hz was used in the study by Mendonça et al. [5]. TENS was applied. The duration of TENS application was 20 min in two studies [5, 7] and 30 min in three of the studies [6, 21, 23]. Stimulation was performed in the first 24 h [7] without preoperative anesthesia [23], starting preoperatively and continuing for the first 3 days postoperatively [6], and in two studies, three times a week postoperatively applied [5, 21] (Table 4).

**Table 4:** Stimulation parameters

Studies	Place of adhesive electrodes	Wave-form	Pulse duration	Frequency	Intensity of stimulation	Treatment duration
**Mendonça, 2017 **[5]	P1 and P2 points	A biphasic and asymmetric	100 µs	100 Hz	The stimulation intensity was raised to the maximum sensory level tolerated by the patient, which was determined by raising the amplitude of the current until obtaining a visible motor contraction and reducing it until the contraction dissipates	Three times per week, on alternate days, during 20 sessions (20 min)
**Ao, 2021 **[6]	Acupuncture points PC6, LI4, and ST36	A dense‑and‑disperse frequency	NS	2/100 Hz	The stimulation intensity was set to mild twitching of the surrounding muscle and individual maximum tolerance	Postoperative 4 and 12 h on the day of surgery, and administrated three times (8 a.m., 2 p.m., and 8 p.m.) daily on postoperative days 1 and 2 (30 min)
**Erden, 2022 **[7]	2 cm from the drain	NS	50–250 ms	85 Hz	The stimulation intensity was increased to a level that would cause a feeling of paresthesia but no pain	Postoperative 11th and 23rd h (20 min)
**Elshinnawy, 2024 **[21]	To the lower edge of the medial tibial condyle and three inches above the medial malleolus	NS	250 s	15 Hz	The stimulation intensity was adjusted to create strong, rhythmic visible muscular contractions under the electrodes (moderately bearable intensity)	Three times per week, for a period of 12 weeks (30 min)
**Lu, 2021 **[23]	Acupuncture points PC6 and CV17	A dense‑and‑disperse frequency	NS	2/10 Hz	The stimulation intensity was identified as the maximal tolerance to the “Teh Chi” sensations of heaviness, numbness, and swelling at the point of stimulation	Before induction of anesthesia (30 min)

*NS* not specified

### Postoperative effects of TENS therapy

#### Pain relief

Only two of the studies included information about pre-treatment pain; Lu et al. reported the number and percentage of patients who experienced preoperative pain at the surgical site, Elshinnawy et al. reported the severity of pain before and after treatment [21, 23]. In all of the studies reviewed, transcutaneous electrical stimulation had a beneficial effect on postoperative pain [5–7, 21, 23]. In Erden et al., a statistically significant difference was found between the intervention and control groups in favor of the intervention group in terms of experiencing less pain in the first 24 h, performing activities such as turning, sitting, repositioning in bed, engaging in out-of-bed activities like walking, sitting on a chair, standing at the sink, falling asleep, and staying asleep (*p* < 0.05). Additionally, it was observed that the number of people who experienced very severe pain in the first 24 h was higher in the control group [7].

In Lu et al.’s study, pain was reported in 30.5% of the combined acupoint group, 35.4% of the single acupoint group, and 39.9% of the sham group in the third postoperative month in patients with breast cancer who underwent TEAS after mastectomy (*p* < 0.05) [23]. At the sixth postoperative month, 22.1% of the combined acupoint group, 36.4% of the single acupoint group, and 34.6% of the sham group reported pain. However, there was no statistical difference between the groups regarding the NRS pain score of patients reporting pain at the postoperative sixth month (*p* > 0.05). Pairwise comparison showed that patients in the combined acupuncture points group had a lower NRS score than the sham group at postoperative three months (*p* < 0.05; risk ratio, 95% CI − 0.56 [− 1.01, − 0.11]). At 6 months postoperatively, the combined acupoint group had a lower pain score than the single acupoint group (*p* < 0.05; risk ratio, 95% CI 0.72 [0.55, 0.93]) and the sham group (*p* < 0.05; risk ratio, 95% CI 0.68 [0.52, 0.89]).

In the study by Ao et al. [6], it was reported that postoperative pain scores were significantly lower in the TEAS group at 12 and 24 h postoperatively compared to the sham group. However, the difference at 4 and 48 h postoperatively was not statistically significant. In the study, opioid analgesic demand and consumption from the PCA pump were significantly lower in the TEAS group compared to the sham group in the postoperative 48 h. When the groups were compared, the incidence of postoperative nausea, vomiting, and headache was significantly lower in the TEAS group. However, there was no statistically significant difference in the incidence of pruritus and dizziness between the two groups [6].

In another study, intraoperative remifentanil consumption was significantly lower in the combined acupuncture TEAS group compared to the sham group (*p* < 0.001, 95% CI − 0.21 [− 0.34, − 0.07]). The first postoperative verbal response (*p* > 0.05) and time to endotracheal extubating (*p* > 0.05) did not differ in the combined acupuncture group and the single acupuncture group compared to the sham group [23].

The NRS pain score at rest (*p* = 0.12) and during coughing (*p* = 0.49) did not show a statistically significant difference between the three groups. There was no statistical difference between the groups regarding postoperative rescue analgesic requests and patient satisfaction scores [23].

Studies were analyzed in terms of pain control in the process of TENS application after breast surgery. In one of the studies, diclofenac 75 mg intramuscularly on postoperative day 0, paracetamol 500 mg tablet twice on postoperative day 1, and paracetamol were administered as needed until discharge [7]. Two of the studies used sufentanil for patient-controlled postoperative analgesia [6, 23]. Two studies did not include information on analgesia application [5, 20] (Table 2). One of the reviewed studies concluded that TENS reduced pain due to chemotherapy-induced diabetic peripheral neuropathy after mastectomy, but the cold application was more effective than TENS [21].

#### Nausea and vomiting

In one study, the incidence of nausea and vomiting after the first postoperative day was 18.4% in the combined acupuncture TEAS group, lower than 29.8% in the single acupoint TEAS group (*p* = 0.004; risk ratio, 95% CI 0.71 [0.53, 0.94]) and 36.2% in the sham group (*p* < 0.001; risk ratio, 95% CI 0.60 [0.45, 0.81]). The nausea and vomiting score in the combined acupuncture group was lower than in both the single acupoint and sham groups (*p* < 0.001). However, further analysis revealed that the incidence of nausea differed between groups (*p* < 0.001), but the incidence of vomiting did not (*p* > 0.05). TEAS is associated with lower analgesic consumption and lower postoperative nausea and vomiting scores in patients undergoing radical mastectomy under general anesthesia [23].

#### Patient satisfaction

Patients in the intervention group with TENS application showed a higher patient satisfaction rate than the control group [7]. Additionally, it was shown that there was a moderate, positive, and meaningful relationship between the level of pain relief participation in treatment and patient satisfaction with analgesia [7]. In one of the included studies, the patient satisfaction scores were similar between the intervention and control groups [23].

#### Immune function

In one study, TEAS protected cellular immune function and reduced postoperative opioid-related side effects [6]. Ao et al. demonstrated that TEAS preserved cellular immunity in mastectomized patients. Although the baseline levels of IL-2, IL-4, and IFN-γ before stimulation were similar between the two groups, serum levels of IL-2, IFN-γ, and IL-2/IL-4 ratio were higher in the TEAS group compared to the sham group at 24 and 48 h postoperatively [6].

#### Quality of life

Although similar to placebo, TENS reduced the intensity of dysesthesia after breast surgery but did not significantly change the quality of life compared to placebo [5].

### Side effect

Only one of the studies reviewed did a patient report skin discomfort at the acupuncture points where the TEAS electrodes were applied [23]. Other studies have not reported any side effects related to electrical stimulation [5–7, 21].

## Discussion

TENS is preferred for pain management worldwide because it is inexpensive, noninvasive, self-administered, and has no potential for toxicity or overdose. TENS can be used in combination with medications without potential interactions, and in many countries, TENS devices can be purchased over the counter from pharmacies or online [29].

This review is the first study to evaluate the clinical efficacy of transcutaneous electrical stimulation after breast surgery. In this review, we examined the effects of TENS application compared to standard treatment or sham TENS on postoperative pain in patients undergoing breast surgery [5–7, 21, 23], patient satisfaction [7, 23], analgesic consumption [6, 23], nausea and vomiting score [5, 7, 23], endotracheal intubation time and first verbal response time after extubating [23], perioperative immunity [6], nerve conduction in patients who develop peripheral neuropathy [21], and cutaneous sensitization in the dermatome and quality of life [5].

A total of five randomized controlled trials met the inclusion criteria; all of this included randomization, but the randomization process was unclear in one of the trials [21]. Performance bias is at the heart of the debate about the quality of studies on TENS. Strategies for assessing the blinding of participants and practitioners have differed in previous reviews, with some reviewers arguing that TENS studies always have a high risk of bias because it is not possible to blind participants to the sensations of TENS as they will know whether they are receiving an active or placebo intervention [9]. Participants could not be blinded except for one of the studies included in this review [5].

The results of this study showed a decrease in VAS scores in the intervention group compared to the control group after TENS application after breast surgery. However, it is seen that the common effect size of the studies in the review is low and does not show statistical significance. This may be due to the heterogeneity of the studies, low sample sizes, and the application of the intervention at different TENS protocols in all studies. Differences in TENS parameters such as frequency (2/10 Hz, 100 Hz), waveforms, pulse duration, and application times in the studies in this review may lead to inconsistent results regarding the analgesic effects of TENS. The differences in the results may have resulted from the lack of a standardized protocol.

The analgesic effect of TENS is explained by the gate control theory, which provides attenuation of nociceptive stimulation of large-diameter afferent fibers in the dorsal horn of the spinal cord [30]. Traditional TENS relieves pain by activating A-α and A-β fibers. TENS also activates the endogenous opioid system. This activation is provided by the high and low-frequency stimuli of TENS [31]. In the studies in which low frequency (2/10 Hz) [23] and burst type (low and high frequency) (2/100 Hz) [6] TEAS were applied, a statistically significant decrease was observed in opioid consumption of patients after mastectomy. In Huang et al.’s study, the application of TEAS at different frequencies was compared, and it was found that 2/100 Hz TEAS reduced intraoperative opioid consumption by 33.5%; significantly decreased extubation time, time to reach discharge criteria, and NRS score immediately after extubation; and reduced anesthetic recovery time, while 100 Hz TEAS reduced PONV morbidity [32]. In a randomized clinical study with a small sample size (*n* = 18) of patients undergoing breast surgery, it was reported that acupuncture TENS application at slow (8–10 Hz) and fast alpha (10–12 Hz) wave frequencies reduced the patients’ pain by 88.4 ± 10.7%, while burst-type TENS pain reduced by 66.3 ± 24.6% [8]. In a meta-analysis of 11 studies with optimal treatment parameters in TENS application, the median frequency was 85 Hz [33]. The studies analyzed in this review show that TENS shows analgesic efficacy at different frequencies. β-endorphin concentrations increase in the bloodstream and cerebrospinal fluid after high or low-frequency TENS application [34, 35]. Increased methionine enkephalin, a delta opioid agonist, and dynorphin A, a kappa opioid agonist, are observed in the lumbar cerebrospinal fluid after treatment of patients with low or high-frequency TENS, respectively [36]. This suggests that different opioids are released at the spinal level with different stimulation frequencies. Thus, different opioid receptors are likely activated to produce analgesia with high or low-frequency TENS. Taken together, these data suggest that various opioids and their receptors may play a role in pain relief by TENS [37]. Postoperative pain should be provided with a multimodal approach as a combination of pharmacological and non-pharmacological treatment methods. When cost and risk/benefit analyses are considered, the literature highlights the advantages of using TENS for non-pharmacological analgesia. The safety profile of TENS is favorable compared with drugs. Side effects are low and contraindications are few [38]. In the postoperative period, TENS often appears to be used to complement standard analgesics for symptomatic treatment in pain management, rather than serving as a stand-alone method. This is thought to be due to the uncertainty in studies evaluating the efficacy of TENS on postoperative pain, which has been ongoing for more than 50 years [38].

The short-term and long-term postoperative effects of TENS application on analgesic efficacy were analyzed, highlighting its impact across different time points. Specifically, TENS application resulted in less pain on the first postoperative day compared to the control and sham groups [6, 7]. Additionally, regarding analgesic consumption, a meta-analysis involving 1350 patients demonstrated that TENS reduced postoperative opioid use by more than 25% [34]. In a prospective, randomized, double-blind, placebo-controlled study (*n* = 74) evaluating the effects of TEAS on the quality of recovery and postoperative analgesia within 24 h after gynecological laparoscopic surgery, compared to the control group, the TEAS group had lower postoperative pain scores and cumulative opioid use (*p* = 0.04) [14]. These findings suggest that TENS provides both immediate and extended benefits in postoperative pain management.

In three of the studies discussed in this review [5, 6, 23], the effects of TENS therapy on postoperative nausea and vomiting have been reported. Mendonça et al. found no reduction in nausea-vomiting scores observed in breast cancer patients who underwent 20 sessions of TENS in the postoperative period [5]. In the studies by Lu et al. and Ao et al., it was reported that nausea-vomiting was less common in patients with TEAS applied to acupuncture points [6, 23].

The anti-PONV effects of acupuncture are believed to result from alterations in the activity of neurochemicals such as endorphins, serotonin, and norepinephrine within the central nervous system. These changes help desensitize the brain’s vomiting center and enhance the body's natural anti-vomiting mechanisms. However, once the vomiting center becomes sensitized, reversing this sensitivity becomes challenging [39]. It is hypothesized that high-frequency TEAS stimulates the early release of a greater amount of neurochemicals, which promptly desensitize the vomiting center. This mechanism may explain why a frequency of 100 Hz demonstrated significant anti-PONV effects [33]. A systematic review and meta-analysis of 26 studies reporting the effects of electrical acupoint stimulation were reported to be effective in reducing postoperative nausea and vomiting, with a moderate to low level of evidence [40]. Viderman et al. reported in a systematic review and meta-analysis on the postoperative effects of TENS in 2265 patients that using TENS reduced postoperative nausea and vomiting, although at a low level of evidence [41]. Another systematic review evaluated nine of the 22 studies on the effect of TEAS on nausea and vomiting, showing beneficial effects compared to standard treatment [11]. TEAS has also been reported to reduce nausea, vomiting, and antiemetic consumption in the sixth postoperative hour [42]. In a non-inferiority randomized controlled study comparing the effectiveness of TEAS and dexamethasone in preventing postoperative nausea and/or vomiting within 24 h after breast surgery, it was noted that TEAS was as effective as dexamethasone [28]. In conclusion, TEAS is recommended as a feasible method to prevent postoperative nausea and/or vomiting.

## Limitations of the review

Some limitations should be noted when interpreting the results of this review. The review’s search strategy and inclusion criteria may carry a risk of publication bias. Focusing on English-language studies only and excluding randomized controlled trial protocols, conference abstracts, and theses risked missing relevant data or studies with conflicting results that may affect the overall findings. Additionally, there was a risk of bias in four of the included studies due to the management of the intervention and incomplete blinding. The lack of blinding is a shortcoming in terms of understanding whether the analgesic efficacy of the application is due to the real or the placebo effect. Moreover, the small sample size of some included studies may affect the generalizability of the results. The main limitation of this review is the limited number of studies included. In addition, the fact that preoperative pain intensity was not given in the studies, which may affect the assessment of postoperative outcomes, was not considered a potential confounder and is among the limitations of the studies.

## Conclusions

This systematic review emphasizes that postoperative TENS application reduces pain intensity, decreases nausea and vomiting, and lowers opioid consumption in breast cancer patients who have undergone surgery. However, some studies were limited in terms of factors such as lack of preoperative pain intensity assessment, small sample sizes, and risks of bias. These limitations indicate that more studies with larger sample sizes, appropriate blinding methods, and more rigorous study designs are needed to strengthen the evidence. Although both the short-term and long-term effects of TENS appear to be beneficial for pain management in a manner consistent with the literature, the generalizability of these effects is limited due to heterogeneous protocols. To increase the level of evidence of the current results and reduce heterogeneity, high-quality studies comparing the effects of standardized TENS protocols are needed. After breast cancer surgery, TENS application can accelerate patients’ recovery by reducing pain. In addition, its effect on nausea and vomiting and its reduction in opioid consumption may increase patient comfort and satisfaction. Due to its cost-effectiveness and ease of use, it allows the treatment to be continued at home. This therapy can be an integral part of patient care and rehabilitation, especially when part of a more comprehensive therapeutic program.

Future research should focus on assessing the severity and characteristics of baseline pain in patients undergoing breast surgery, as well as exploring the long-term effects of TENS on pain and other postoperative outcomes. Additionally, large-scale clinical trials, including sham TENS groups, are necessary to confirm the clinical utility of this technique. Studies should also consider using devices that terminate the electrical current after a brief period to ensure proper blinding.

## Supplementary Information

Below is the link to the electronic supplementary material.

Supplementary file1 (DOCX 32 KB)

## Data Availability

Data sharing is not applicable to this article as no new data were created or analysed in this study.
